# Supplementary material for: Sodium Ion Pre-Intercalation of δ-MnO2 Nanosheets for High Energy Density Aqueous Zinc-Ion Batteries
Source: Nanomaterials (Basel). 2023 Mar 16;13(6):1075. doi: 10.3390/nano13061075 (PMC10057495; doi:10.3390/nano13061075)
Supplement: Supplementary file 1 [file nanomaterials-13-01075-s001.zip › nanomaterials-2252947-supplementary.pdf]

nanomaterials

MDPI

Article

Sodium Ion Pre-Intercalation of δ-MnO_2 Nanosheets for High Energy Density Aqueous Zinc-Ion Batteries

Yuanhao Ding [1], Weiwei Xue [1], Kaihao Chen [1], Chenghua Yang [1], Qi Feng [1], Dezhou Zheng [1,*], Wei Xu [1], Fuxin Wang [1,*] and Xihong Lu [1,2]

1 School of Applied Physics and Materials, Wuyi University, Jiangmen 529020, China
2 MOE of the Key Laboratory of Bioinorganic and Synthetic Chemistry, The Key Lab of Low-Carbon Chem & Energy Conservation of Guangdong Province, School of Chemistry, Sun Yat-sen University, Guangzhou 510275, China
* Correspondence: zhengdz19@126.com (D.Z.); wangfux91@126.com (F.W.)

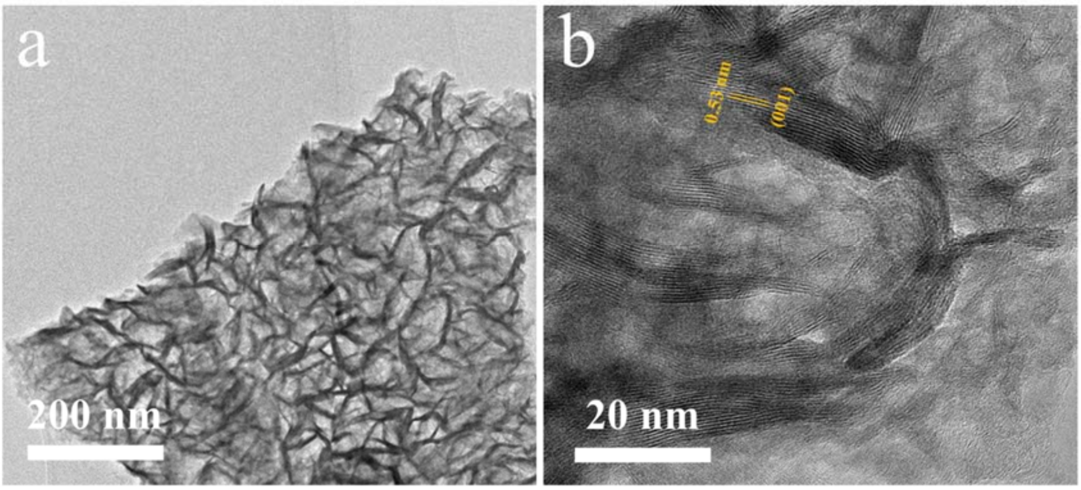

Figure S1. (a) and (b) TEM and HRTEM images of MnO_2.

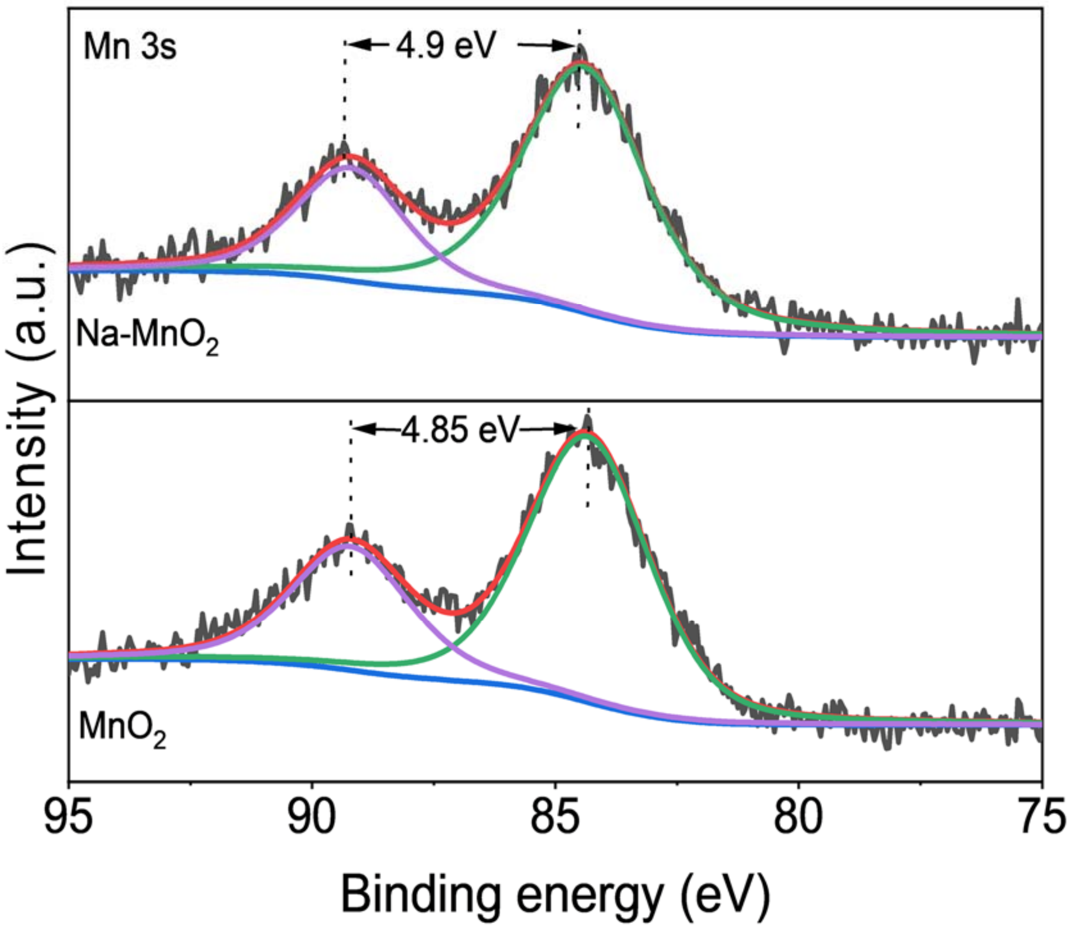

Figure S2. Mn 3s spectra of the MnO_2 and Na-MnO_2 sample.

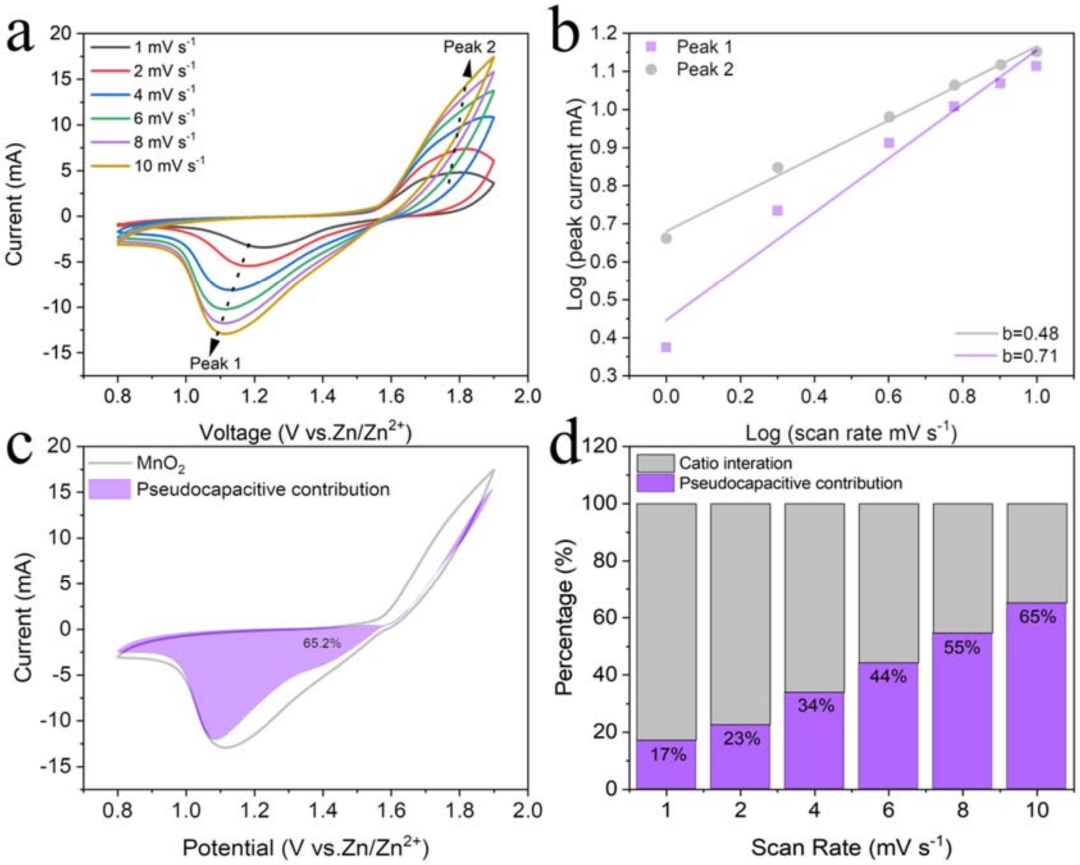

Figure S3. (a) CV curves of MnO_2 electrode at different scan rates. (b) log (peak current) versus log (scan rate) plots of each peak. (c) Capacitive contribution (inset) and diffusion contribution of MnO_2 electrode at 10 mV s^{-1}. (d) The capacitive contributions at scan rates of 1, 2, 4, 6, 8 and 10 mV s^{-1}.
